# Spread of Carbapenem Resistance by Transposition and Conjugation Among *Pseudomonas aeruginosa*

**DOI:** 10.3389/fmicb.2018.02057

**Published:** 2018-09-05

**Authors:** Anneke van der Zee, W. Bart Kraak, Arjan Burggraaf, Wil H. F. Goessens, Walter Pirovano, Jacobus M. Ossewaarde, Jan Tommassen

**Affiliations:** ^1^Laboratory of Medical Microbiology, Molecular Diagnostics Unit, Maasstad Hospital, Rotterdam, Netherlands; ^2^Erasmus University Medical Center, Rotterdam, Netherlands; ^3^BaseClear, Leiden, Netherlands; ^4^Section Molecular Microbiology, Department of Biology, Faculty of Science, Utrecht University, Utrecht, Netherlands

**Keywords:** *Pseudomonas aeruginosa*, genome sequence, carbapenem resistance, VIM-2, integron, transposon, conjugation, integrative and conjugative element

## Abstract

The emergence of carbapenem-resistant *Pseudomonas aeruginosa* represents a worldwide problem. To understand the carbapenem-resistance mechanisms and their spreading among *P. aeruginosa* strains, whole genome sequences were determined of two extensively drug-resistant strains that are endemic in Dutch hospitals. Strain Carb01 63 is of O-antigen serotype O12 and of sequence type ST111, whilst S04 90 is a serotype O11 strain of ST446. Both strains carry a gene for metallo-β-lactamase VIM-2 flanked by two *aacA29* genes encoding aminoglycoside acetyltransferases on a class 1 integron. The integron is located on the chromosome in strain Carb01 63 and on a plasmid in strain S04 90. The backbone of the 159-kb plasmid, designated pS04 90, is similar to a previously described plasmid, pND6-2, from *Pseudomonas putida*. Analysis of the context of the integron showed that it is present in both strains on a ∼30-kb mosaic DNA segment composed of four different transposons that can presumably act together as a novel, active, composite transposon. Apart from the presence of a 1237-bp insertion sequence element in the composite transposon on pS04 90, these transposons show > 99% sequence identity indicating that transposition between plasmid and chromosome could have occurred only very recently. The pS04 90 plasmid could be transferred by conjugation to a susceptible *P. aeruginosa* strain. A second class 1 integron containing a gene for a CARB-2 β-lactamase flanked by an *aacA4′-8* and an *aadA2* gene, encoding an aminoglycoside acetyltransferase and adenylyltransferase, respectively, was present only in strain Carb01 63. This integron is located also on a composite transposon that is inserted in an integrative and conjugative element on the chromosome. Additionally, this strain contains a frameshift mutation in the *oprD* gene encoding a porin involved in the transport of carbapenems across the outer membrane. Together, the results demonstrate that integron-encoded carbapenem and carbapenicillin resistance can easily be disseminated by transposition and conjugation among *Pseudomonas aeruginosa* strains.

## Introduction

*Pseudomonas aeruginosa* is an opportunistic gram-negative pathogen causing acute and chronic infections in hospitalized and immune-compromised patients (Kerr and Snelling, 2009). *Pseudomonas* species are known to have evolved from a wide variety of environments and are highly adaptable (Silby et al., 2011). In recent years, extensively drug-resistant *P. aeruginosa* present a globally increasing problem in hospital environments (Edelstein et al., 2013; Wright et al., 2015). *P. aeruginosa* can rapidly become resistant to antibiotics due to various mechanisms (Oliver et al., 2015), including chromosomal mutations leading to inducible hyper-production of chromosomal AmpC β-lactamase, overexpression of efflux pumps, and/or reduced membrane permeability. Such mutations together are referred to as the mutational resistome (Lopéz-Causapé et al., 2018). Additionally, horizontal transfer of mobile genetic elements, such as integrons, transposons, or plasmids, can confer resistance mechanisms. The increasing prevalence of metallo-β-lactamases (MBLs), such as VIM or IMP, can be caused by horizontal acquisition of integrons, which are often found to contain also genes encoding aminoglycoside resistance. As a result of frequent acquisition of mobile DNA elements, the genome of *Pseudomonas* can be divided into a core genome and an accessory genome (Battle et al., 2009; Oliver et al., 2015). The population structure therefore was termed non-clonal epidemic, with a high recombination frequency between isolates (Kidd et al., 2012).

Carbapenemase-producing *P. aeruginosa* are often only susceptible to colistin (Edelstein et al., 2013; Kos et al., 2015), thus limiting therapeutic options to treat infected patients. Worldwide, carbapenemase-producing *P. aeruginosa* were assigned to successful clonal complexes (CCs) by multi-locus sequence typing (MLST) and O-antigen serotyping (Thrane et al., 2015). CCs 111 and 235 are considered responsible for the worldwide dissemination of extensively drug-resistant lineages which are of the serotypes O12 and O11, respectively (Thrane et al., 2015). The prevalence of ST111 in the Netherlands was previously described (Van der Bij et al., 2012), and this sequence type was also involved in outbreaks in the United Kingdom (Breathnach et al., 2012). ST235/O11 is more prevalent in Eastern European countries, including Russia (Oliver et al., 2015).

For several years, multidrug-resistant, VIM-producing *P. aeruginosa* were isolated in several hospitals in the Rotterdam area, The Netherlands. In the Erasmus University Medical Centre, two different genotypes predominate (Van der Bij et al., 2011), one of which, an ST111 clone, also dominates in the Maasstad Hospital as well as in many other hospitals in the Netherlands (Van der Bij et al., 2012). Both strains persist and spread through the hospitals via the sinks and drains in spite of hypochlorite treatment. Here, we analyzed representative isolates of both genotypes, which were subjected to whole genome sequencing to study their genetic background. To gain insight into the mobilization of integrons, we analyzed the context of the integrons in detail.

## Materials and Methods

### Ethics Statement

*P. aeruginosa* strains were selected under designated names and were not related to patients. According to the Dutch regulation for research with human subjects, no medical or ethical approval was required to conduct this study. The regional medical ethics committee (Toetsingscommissie Wetenschappelijk Onderzoek Rotterdam e.o.) waived the need for informed consent and approved the study (L201586), in agreement with national law by the Federation of Dutch Medical Scientific Societies^1^.

### Strains

*P. aeruginosa* strain Carb01 63 is a representative of the dominant ST111 genotype isolated from drains and sinks of the Intensive Care Unit at Maasstad Hospital, Rotterdam. Strain S04 90 is a representative of a distinct genotype, i.e., ST446, and was isolated from a patient in Erasmus University Medical Center. Strain PAO1 (Holloway, 1955) was used in conjugation experiments.

### Whole Genome Sequencing

Genomic DNA libraries for the Illumina and PacBio platforms were generated and sequenced at BaseClear B.V. (Leiden, Netherlands). For Illumina sequencing, high-molecular-weight genomic DNA was used as input for library preparation using the Illumina TruSeq library preparation kit. Briefly, the genomic DNA was fragmented by nebulization and subjected to end repair, A-tailing, ligation of adaptors including sample-specific barcodes, and size selection. After PCR enrichment, the resultant library was checked on a Bioanalyzer (Agilent) and quantified. The libraries were multiplexed, clustered, and sequenced on an Illumina HiSeq 2000 instrument with paired-end protocol. For PacBio sequencing, high-molecular-weight genomic DNA was sheared to fragments of about 10 kb in length using G-tubes (Covaris) and further processed into a PacBio sequencing library using the standard protocols (Pacific Biosciences). The resulting PacBio library was checked on a BioAnalyzer (Agilent), quantified and sequenced on a PacBio RSII instrument.

Illumina FASTQ sequence files were generated using the Illumina Casava pipeline version 1.8.3. Initial quality assessment was based on data passing the Illumina Chastity filtering. Subsequently, reads containing adapters and/or PhiX control signal were removed using an in-house filtering protocol. The second quality assessment was based on the remaining reads using the FASTQC quality control tool version 0.10.0.

The long read data collected from the PacBio RS instrument were processed and filtered using the SMRT Analysis software suite. The Continuous Long Read (CLR) data were filtered by Read length (>50), Sub read length (>50), and Read quality (>0.75).

The quality of the Illumina FASTQ sequences was enhanced by trimming off low-quality bases using the “Trim sequences” option of the CLC Genomics Workbench version 7.5.1. The quality-filtered sequence reads were puzzled into a number of contig sequences using the “*De novo* assembly” option of the CLC Genomics Workbench version 7.5.1. The optimal *k*-mer size was automatically determined using KmerGenie (Chikhi and Medvedev, 2014). The contigs were linked and placed into super-scaffolds based on the alignment of the PacBio CLR reads. Alignment was performed with BLASR (Boetzer and Pirovano, 2014). From the alignment, the orientation, order and distance between the contigs were estimated using the SSPACE-LongRead scaffolder version 1.0 (Boetzer and Pirovano, 2014). Final adjustments were manually made based on the assembly graph. The gapped regions within the super-scaffolds were (partially) closed in an automated manner using GapFiller version 1.10 (Boetzer and Pirovano, 2012). The method takes advantage of the insert size between the Illumina paired-end reads.

### Genome Analysis

All sequences were automatically annotated using the NCBI Prokaryotic Genomes Automatic Annotation Pipeline (PGAAP)^2^ followed by the Rapid Annotations using Subsystems Technology (RAST) server (Aziz et al., 2008). Prophages were assigned by Phage Search Tool (PHAST) (Zhou et al., 2011). The CRISPRFinder (Grissa et al., 2007) was used to search the genomes for possible CRISPR fragments. Restriction-modification systems were analyzed by submission of PacBio data in REBASE (Roberts et al., 2015). Multi-locus sequence types were assigned^3^ for both strains. O-antigen types of both strains were determined on the BLAST server of NCBI, where the whole genome sequences were aligned against the different O-antigen sequences of O1-O20. IS finder^4^ was used to identify IS elements (Siguier et al., 2006).

### Conjugation

Strain PAO1 was consecutively exposed to increasing concentrations of fosfomycin (FOS) to select for mutants with minimum inhibitory concentration (MIC) > 256 μg ml^-1^, needed for counter selection. After overnight growth, 5 μl of FOS-resistant PAO1 was mixed with 5 μl of strain S04 90. Subsequently, the suspension was plated on MacConkey agar plates containing FOS (150 μg ml^-1^), and five disks each containing 10 μg meropenem (MER) were placed on the plates. The selected colonies resistant to FOS and MER were investigated for the presence of the VIM gene by PCR (Van der Zee et al., 2014). VIM-positive colonies were typed by amplified fragment length polymorphism (AFLP) to verify if they were PAO1 derivatives. VITEK2 (Biomérieux, Marcy l’Etoile, France) analysis was used to determine antibiotic resistance in donor, recipient and transconjugant strains.

### AFLP Typing

Amplified fragment length polymorphism typing was performed essentially as described previously (Van der Zee et al., 2003), except that primers were labeled at the 5′ end with Yakima Yellow. Fragments were analyzed by capillary electrophoresis in an ABI3500 instrument and compared to GeneScan^TM^ 600 LIZ^®^ Size Standard v2.0 (Life Technologies, Bleiswijk, Netherlands). Peak patterns were converted to banding patterns using Bionumerics v7.6 (Applied Maths, St Martens Latem, Belgium). Cluster analysis of the fingerprints was performed by Unweighted Pair Group Method with Arithmetic mean (UPGMA).

### Nucleotide Sequence Accession Numbers

The nucleotide sequences of the Carb01 63 chromosome, the S04 90 chromosome, and the pS04 90 plasmid were deposited in Genbank under accession numbers CP011317.1, CP011369.1, and CP011370.1, respectively. Complete assemblies of Carb01 63 and S04 90 are filed under assembly numbers ASM98182v1 and ASM98850v1, respectively.

## Results

### Bacterial Isolates

Genotyping by multi-locus variable-number tandem-repeat analysis (MLVA) revealed two separate clusters of strains among the VIM-2 MBL-producing *P. aeruginosa* in the hospitals in the Rotterdam area (Van der Bij et al., 2011). MLST revealed that the main cluster consisted of ST111 strains, whilst a minor cluster contains strains of ST446 (Van der Bij et al., 2012). A representative of each of these clusters was elected for genome sequence analysis, i.e., strains Carb01 63 and S04 90 of ST111 and ST446, respectively. VITEK2 analysis showed that both strains are resistant to most antibiotics commonly used to treat *P. aeruginosa* infections but are sensitive for colistin (**Table 1**).

**Table 1:** Antibiograms of strains Carb01 63, S04 90, PAO1, and a transconjugant of PAO1 carrying pS04 90.

Antibiotics	Carb01 63	S04 90	PAO1	PAO1/pS04 90^b^
	MIC^a^	MIC^a^	MIC^a^	MIC^a^
Piperacillin/Tazobactam	≥128	≥128	8	≥128
Ceftazidime	16–32	≥64	4	≥64
Gentamicin	≥16	≥16	≤1	≤1
Tobramycin	≥16	≥16	≤1	≥16
Colistin	≤0.5	≤0.5	≤0.5	≤0.5
Ciprofloxacin	≥4	≥4	≤0.25	≤0.25
Meropenem	≥16	8-≥ 16	1	≥16
Imipenem	≥16	≥16	2	≥16
Cefepime	≥64	≥64	2	16
Amikacin	≥64	ND	ND	ND
Fosfomycin	32	64	ND	ND

*^*a*^MIC is given in μg ml^-*1*^. ^*b*^Increased MIC values of the transconjugant relative to the recipient strain are marked in gray*.

### Genome Analysis

The complete nucleotide sequence of the Carb01 63 genome was recovered by *de novo* hybrid assembly. To overcome sequencing difficulties as a result of large repeats in the genome of S04 90, the sequences were aligned with that of UCBB-PA14 (Lee et al., 2006) which resulted in a single sequence of the chromosome with five gaps of in total 152 bp. This strain was found to contain a large plasmid of 159,187 bp, designated pS04 90. The plasmid has a GC content of 57.7% (**Table 2**), which is substantially lower than the average chromosomal GC content of *P. aeruginosa.* The strains carry different O-antigens, i.e., O12 in Carb01 63 and O11 in S04 90 (**Table 2**). Bacterial defense systems against the uptake of foreign DNA are CRISPR/Cas and restriction-modification systems. No genes for functional CRISPR/Cas systems were observed in the chromosome of either isolate, but the plasmid pS04 90 encodes a putative CRISPR (**Table 2**). Carb01 63 contains type I, II, and III restriction-modification systems, and two unique type I target recognition domains could be identified. S04 90 contains type I and II restriction-modification systems. Plasmid pS04 90 also contains a type II system. Bacteriophage searches revealed the presence of nine prophages, of which four questionable, in Carb01 63 and of 11 prophages, of which four questionable, in S04 90 (**Table 2**). These high numbers of prophages might be related to the absence of CRISPR/CAS systems on the chromosomes of the strains.

**Table 2:** Characteristics of the chromosomes of Carb01 63 and S04 90 and pS04 90.

	*P. aeruginosa*	*P. aeruginosa*	Plasmid
Characteristics	Carb01 63	S04 90	pS04 90
Genome size (bp)	7,497,593	7,099,963	159,187
GC (%)	65.60	66.01	57.73
Genome coverage	98x	97x	97x
No. genes	7071	6889	160
Coding sequences	6986	6720	160
rRNAs	12 (5S, 16S, 23S)	12 (5S, 16S, 23S)	0
tRNAs	65	65	0
Non-coding RNAs	8	1	0
Pseudo genes	76	91	-
CRISPR/Cas systems	0	0	1 (putative)
MLST type	ST111	ST446	-
*O*-antigen	O12	O11	-
Phages (questionable)	9 (4)	11 (4)	0

Genes for all major virulence factors were found in the genomes of Carb01 63 and S04 90, including alkaline protease AprA, which is a substrate for the type I protein secretion system (T1SS), and elastase LasB, exotoxin A, and the haemolytic and non-haemolytic phospholipases C PlcH and PlcN, which are substrates of the T2SS (Bleves et al., 2010). The T3SS substrates ExoS and ExoU are mutually exclusive and predominantly found in invasive and cytotoxic *P. aeruginosa* strains, respectively (Bleves et al., 2010). Strain Carb01 63 contains an *exoS* gene (locus tag YQ19_07370), whilst an *exoU* gene (locus tag YH69_22740) was found in strain S04 90.

Comparison of the nucleotide sequences of the chromosomes of Carb01 63 and S04 90 revealed 99% identity with query coverage Carb01 63/S04 90 of 88% and S04 90/Carb01 63 of 92% and only minor differences in size and GC content (**Table 2**). Based on the dendrogram generated by genomic BLAST^5^, these sequences are quite distinct (**Supplementary Figure S1**). Carb01 63 belongs to a large clade of 152 leaves which is represented by P17_North_West_14_VIM_2_03_10, whilst S04 90 belongs to a clade of 34 leaves which is represented by the lineage of 468_PAER. Both clades contain related strains of medical origin mostly from the United Kingdom and France. Schematic representations of both genomes and of some well-described *P. aeruginosa* genomes were made by progressive Mauve (Darling et al., 2010) to indicate the similarities and differences (**Supplementary Figure S2**).

### Characteristics of the Integrons in Strains Carb01 63 and S04 90

**FIGURE 1:**
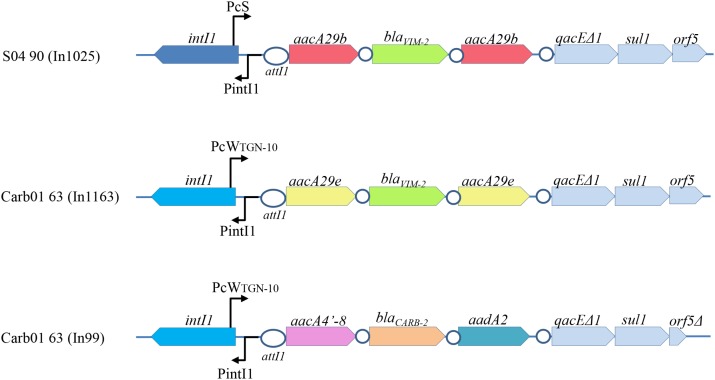
Schematic representation of the integrons of Carb01 63 and S04 90. The gene cassette promoters (Pc) are different. PcS represents a strong promoter, whereas PcW_TGN-10_ represents a promoter that is considerably weaker due to nucleotide substitutions in the –10 and –35 regions, which are, however, partially compensated by a C to G substitution upstream of the –10 region, resulting in an extended –10 motif (Jové et al., 2010). These differences also affect the primary structures of the integrases encoded by the *intI1* genes (dark and bright blue). Cassettes with the same nucleotide sequences are indicated with the same color. No differences were observed in the 3′ conserved sequences, except that *orf5* in In99 is truncated. The integration sites *attI1* and *attC* are indicated by ovals and circles, respectively.

Outbreak strains in the Rotterdam area were reported to contain a *bla*_V IM-2_ gene on an integron (Van der Bij et al., 2011). Consistently, *bla*_V IM-2_-containing class 1 integrons were found in the genome sequences of strains Carb01 63 and S04 90 and they were designated In1163 and In1025, respectively (**Figure 1**). In Carb01 63, the integron was found on the chromosome, whilst it was present on the plasmid in S04 90. The sequences of integrons In1025 and ln1163 are closely related (**Figure 1**) and very similar but not identical to that of In59 (Poirel et al., 2001). Small differences were observed in the gene cassette promoter *Pc* and in the *aacA* genes flanking *bla*_V IM-2_ and conferring aminoglycoside resistance (**Figure 1**). Differences in the *Pc* promoter affect promoter strength and, because the *Pc* promoter is located within the *intI1* coding sequence, also the integron-excision activity of the encoded integrase (Jové et al., 2010). Whilst In1025 contains two *aacA29b* genes, these genes are replaced by *aacA29e* genes in In1163 (**Figure 1**). With respect to the *aacA29* genes, both strains are also different from other analyzed outbreak strains in the Rotterdam area, which were all (*n* = 25) reported to contain *aacA29a* and *aacA29b* genes upstream and downstream of the *bla*_V IM-2_ gene, respectively (Van der Bij et al., 2012). The *aacA29e* genes in In1163 are different from previously described *aacA* genes and their products differ from those of the *aacA29b* genes by a single F41L amino-acid substitution. Although the function of the newly found *aacA29e* genes on the integron of Carb01 63 was not determined, *aacA29a* and -*29b* are known to cause decreased susceptibility to amikacin and to tobramycin, but not to gentamicin (Poirel et al., 2001).

Besides the *bla*_V IM-2_-containing integron, Carb01 63 contains a second class 1 integron, designated In99, containing a *bla*_CARB-2_ gene (**Figure 1**). The *bla*_CARB-2_ gene is flanked by an *aacA4′-8* gene [a.k.a. *aac(6′)-Ib*] encoding an aminoglycoside 6′-*N*-acetyltransferase and an *aadA2* gene encoding an aminoglycoside-3^′′^-adenylyltransferase, which is associated with resistance to streptomycin and spectinomycin. Class 1 integrons with such cassette composition were previously described in *P. aeruginosa* isolates from Portugal (Caetano et al., 2007).

### Genetic Context of the *bla*_V IM-2_ Containing Integrons

**FIGURE 2:**
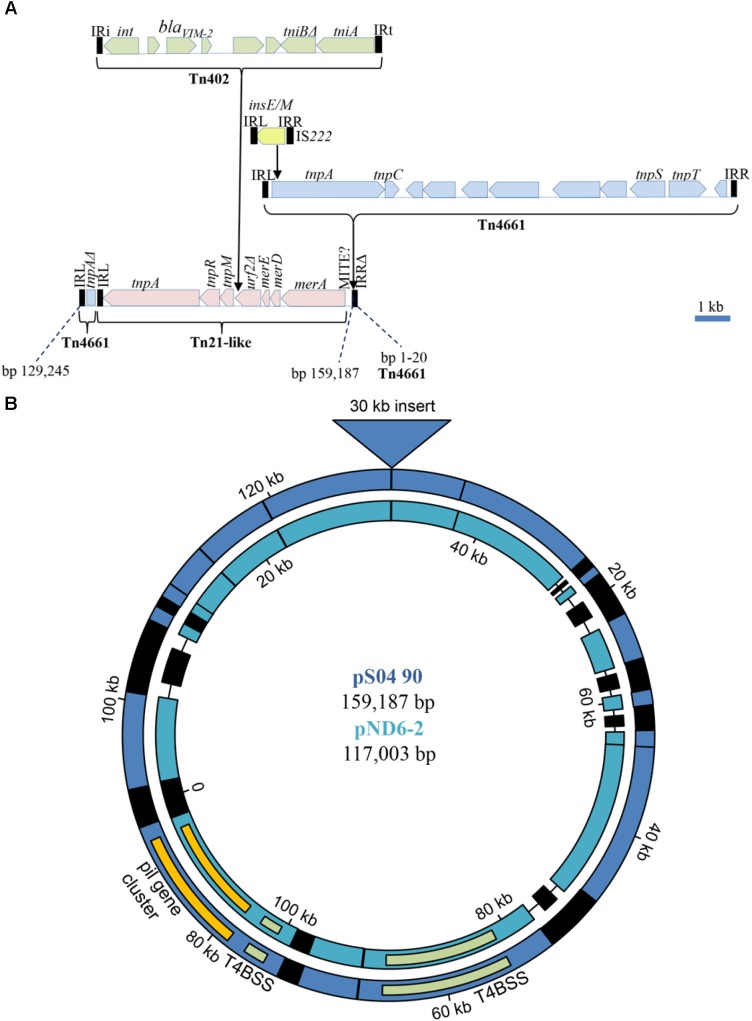
Genetic context of the *bla*_V IM-2_ containing integron on pS04 90. **(A)** Composition of a mosaic 30 kb transposon containing the integron with the *bla*_V IM-2_ gene. Open reading frames of different transposable elements are indicated by different colors. MITE?, 332-bp inserted element possibly representing a MITE. **(B)** Comparison of pS04 90 (blue) with pND6-2 (sea-green). Regions of homology are indicated by color. Non-homologous regions are black and deletions are indicated by a line. The positions of the *pil* gene cluster (yellow) and the genes involved in the type IVB secretion system (T4BSS) (green) are indicated in both plasmids. The large insertion, detailed in **(A)**, is indicated by a triangle at the top. The dissimilar regions (black) around 20, 90, and 105 kb in pS04 90, encode, amongst others, a toxin/antitoxin addiction module, an *O*-antigen transacetylase OafA, and CRISPR-related proteins, respectively. Most of the proteins putatively encoded by the dissimilar regions are hypothetical proteins and several transposases/integrases.

In spite of the different genomic location of the *bla*_V IM-2_-containing integrons in Carb01 63 and S04 90, i.e., on the chromosome and on a plasmid, respectively, they are both located on a ∼30-kb DNA fragment with very high sequence similarity between the strains. On the 159,187-bp plasmid pS04 90, this segment covers nucleotides 129,245–159,187 plus 1–20 (**Figure 2**). The integron is contained in a Tn*402* transposon (Gillings, 2017) that is bounded by 25-nt inverted repeats (IR) (**Figure 2A**). This transposon is immobilized as the *tni* transposition module is incomplete with *tniQ* and *tniC* genes being absent. The Tn*402* transposon is inserted into a Tn*21*-like transposon between two open reading frames, designated *tnpM* (locus tag YH69_34320) and a truncated *urf2* (locus tag YH69_34365). Presumably, these two open reading frames are derived from a single gene, designated *urf2M* that was split by the Tn*402* insertion (Liebert et al., 1999). Insertion resulted in a 5-nt (5′-TCCAT-3′) duplication of the target site. The Tn*21*-like transposon contains several genes involved in conferring mercury resistance but the locus is incomplete as the essential *merP, merT*, and *merR* genes are deleted (**Figure 2A**). This deletion also covers the IRR of the Tn*21* transposon, which is, therefore, immobilized. The Tn*21*-like transposon is contained within a severely disrupted Tn*4661* transposon the remnants of which are an intact 47-nt IRL, the 5′ end of the *tnpA* gene encoding the transposase, and an incomplete IRR that covers only 20 of the 47 nt of a complete IRR (**Figure 2A**). Also inserted in this transposon is a complete Tn*4661* (**Figure 2A**), which has >99% sequence identity with Tn*4661* of *P. aeruginosa* plasmid RMS148 (Yano et al., 2013). Tn*4661* is often found inserted in chromosomes of *P. aeruginosa*, e.g., one copy is found on the chromosome of S04 90, whilst strain S86968 (Genbank accession number CP008865.2) contains two copies. In plasmid pS04 90, this transposon contains an IS*222* insertion element of 1237 bp, containing two overlapping ORFs encoding InsE and the transposase InsM that is generated by translational frameshifting at an (A)_6_G site (Kropinski et al., 1994) (**Figure 2A**). This element is inserted with a 3-nt target site duplication (5′-TAC-3′) into codon 11 of the *tnpA* transposase gene of the Tn*4661* transposon and, probably, prevents expression of this gene. The inserted complete Tn*4661* is separated from the *merA* gene of Tn*21* by a 332-bp element that is bounded by 29-nt perfect inverted repeats (5′-GTTGTGGGATGCAAATAAAGTTTCATCCT-3′). Since three copies of this element are found at different positions in the chromosome of strain Carb01 63, it might be a replicative transposable element, but it does not contain a discernible transposase gene. Probably, it represents a hitherto undescribed miniature inverted-repeat transposable element (MITE), which are non-autonomous mobile elements found in both eukaryotes and prokaryotes (Delihas, 2008). The entire 30-kb composite transposon extending from the IRL to the incomplete IRR of the disrupted Tn*4661* transposon is inserted into a plasmid with a core that shows high similarity with the 117-kb plasmid pND6-2 from *Pseudomonas putida* (Li et al., 2013) with a query coverage and sequence identity of 78 and 95%, respectively (**Figure 2B**). The transposon is inserted into a gene corresponding to *orf042* of the pND6-2 plasmid encoding a large hypothetical protein (Li et al., 2013). Consequently, this gene is split into two pseudogenes with locus tags YH69_34300 and YH69_ 33605 on pS04 90, and the insertion resulted in a target site duplication of five nucleotides (5′-TGTTC-3′).

In strain Carb01 63, the complete 30-kb fragment described above, except for the inserted IS*222* element and the incomplete IRR of the disrupted Tn*4661*, is present on the chromosome (nt 3,707,795–3,736,497). The IS*222* element is found on six other chromosomal sites in the Carb01 63 chromosome. As the *tnpA* gene of the complete Tn*4661* is not disrupted, an active transposase can be produced and the entire 30-kb element may constitute an active composite transposon extending from the IRL of the disrupted Tn*4661* to the IRR of the complete Tn*4661*. Insertion of this composite transposon into the Carb01 63 chromosome, has split a gene putatively encoding a MOSC domain-containing molybdenum cofactor sulfurase into two pseudogenes, one of which (the 5′ end) was not annotated and the other (the 3′ end) was annotated with locus tag YQ19_17550. Insertion resulted in a 5-nt (5′-ATGGA-3′) duplication of the target site. Thus, it can be inferred that the complete composite transposon, including the integron with *bla*_V IM-2_, was acquired by transposition.

### pS04 90 Is a Conjugative Plasmid

pND6-2 is a conjugative plasmid that was reported to mobilize a co-resident plasmid from *P. putida* to *Escherichia coli* (Li et al., 2013). Like pND6-2, pS04 90 carries genes encoding an *icm*/*dot* type IVB secretion system and type IV pili, suggesting it might also be a conjugative plasmid (**Figure 2B**). To investigate whether indeed pS04 90 is transferrable, a conjugation experiment was performed using a FOS-resistant derivative of strain PAO1 as the recipient. After conjugation, seven colonies that were resistant to both MER and FOS were further analyzed. These seven strains, which were all positive for *bla*_V IM-2_ in PCR analysis, were genotyped by AFLP. Two strains showed a similar AFLP profile as PAO1 (see **Supplementary Figure S3** for an example) and are therefore regarded as transconjugants. The other five strains resembled the AFLP profile of S04 90 (data not shown) and are, therefore, presumably spontaneous FOS-resistant mutants of this isolate. FOS-resistant strain PAO1 and both transconjugants were analyzed by VITEK, which confirmed the susceptibility of the PAO1 strain to most antibiotics tested and multidrug resistance of the transconjugants (**Table 1**). Repeated attempts to transfer the plasmid to *E. coli*, either by conjugation or by electroporation, failed indicating that it has a narrow host range.

### Genetic Context of the *bla*_CARB-2_-Containing Integron

**FIGURE 3:**
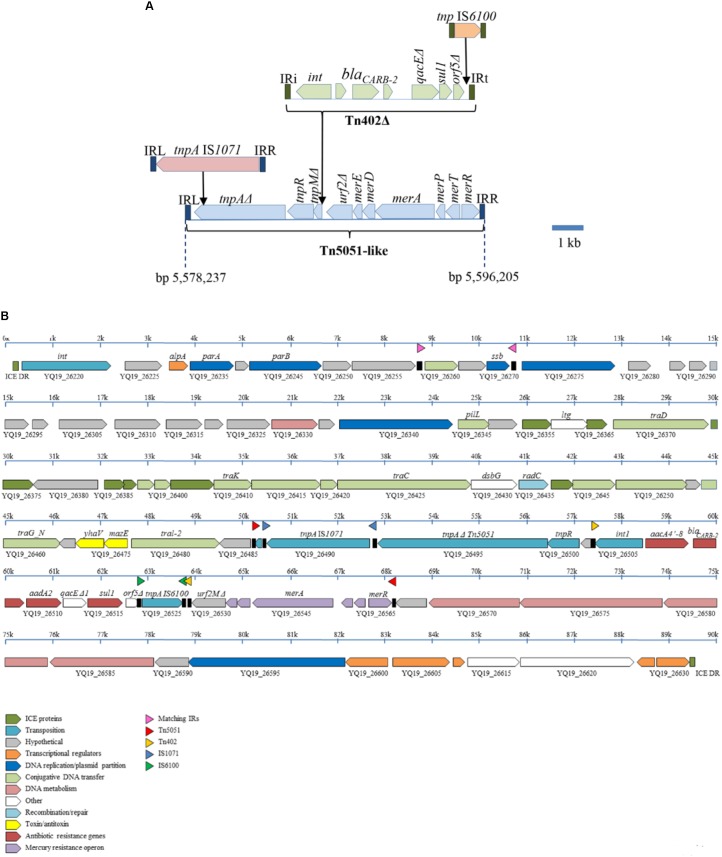
Genetic context of the *bla*_CARB-2_-containing integron in Carb01 63. **(A)** Composition of a mosaic 18-kb transposon containing the integron with the *bla*_CARB-2_ gene. Open reading frames of different transposable elements are indicated by different colors. **(B)** Composition of the ICE into which the composite transposon depicted in **(A)** is inserted. The ICE is bounded by 20-bp direct repeats (ICE DR). ORFs encoding proteins of different functional classes are indicated by different colors as outlined in the inset at the bottom. Inverted repeats are indicated by black boxes in between the ORFs with a colored triangle above.

The In*99* integron with the *bla*_CARB-2_ gene (**Figure 1**) is contained in a severely disrupted Tn*402* transposon that is bounded by 25-nt inverted repeats (**Figure 3A**). This transposon lacks the entire *tni* transposition module. The *orf5* gene of the integron is disrupted by the insertion of an IS*6100* element (**Figure 3A**). The defective Tn*402* is inserted in a Tn*5051*-like transposon, which, apart from the insertion of the Tn*402* and of the IS*1071* element described below, shows high sequence similarity with other Tn*5051*-like transposons, such as Tn*AO22* from *Achromobacter* sp. AO22 (Ng et al., 2009) with only 15 single-nucleotide polymorphisms (SNPs) over an 8230-bp sequence. Like in the Tn*21* transposon described above, the Tn*402* is inserted in the *urf2M* gene but more toward the 3′ end of the gene, i.e., at a position identical to a previously reported integron-insertion site in Tn*5051* (Toleman et al., 2003). Insertion resulted in a 5-nt (5′-GAGTC-3′) duplication of the target site. The genes that are essential for mercury resistance, i.e., *merR, merT, merP*, and *merA*, are complete in the Tn*5051* transposon, and they have indeed been shown to confer mercury resistance in the case of Tn*AO22* (Ng et al., 2009). The *tnpA* gene of the Tn*5051* transposon is truncated by the insertion of an IS*1071* element (**Figure 3A**), which probably renders the Tn*5051* TnpA inactive. The Tn*5051* is bounded by 38-nt inverted repeats and inserted into a gene encoding a hypothetical protein with a DUF4158 domain that is thereby split into two pseudogenes with locus tags YQ19_26565 and YQ19_26485. Insertion resulted in a 5-nt (5′-CTCAA-3′) target site duplication. This disrupted gene is situated within an integrative and conjugative element (ICE), which is integrated in a tRNA-*gly* gene (locus tag YQ19_26215). This 89,494-bp ICE is flanked by two 20-bp direct repeats, corresponding to the 3′ end of the tRNA-*gly* gene and representing the *attL* and *attR* sites (**Figure 3B**). With only very few SNPs, the core composition of the ICE is almost identical to that of the *P. aeruginosa* genomic island PAGI-16 of strain KMU11, but the cassette composition of the integron of this Korean ST235 isolate is different (Hong et al., 2016).

### Mutational Resistome

Besides by the acquisition of genes by horizontal gene transfer, antibiotic susceptibility can be decreased by mutations in the core genome, together constituting the mutational resistome (Lopéz-Causapé et al., 2018). Several mutations are present in the core genome of strains Carb01 63 and S04 90 that likely contribute to the observed resistance phenotypes. The porin OprD mediates the diffusion of carbapenems across the outer membrane, and disruption of the *oprD* gene or downregulation of its expression represents an important carbapenem-resistance mechanism (Pirnay et al., 2002; Lister et al., 2009; Ruiz-Martínez et al., 2011; Cabot et al., 2016). In strain Carb01 63, the *oprD* gene (locus tag YQ19_24920) is disrupted by an 11-bp deletion leading to a frame-shift. This mutation likely contributes to the high level of resistance of the strain to carbapenems. In strain S04 90, the *oprD* gene contains nine mutations leading to amino-acid substitutions relative to OprD of strain PAO1. This variant is identical to variant T1-IV described previously (Ocampo-Sosa et al., 2012), which is not associated with resistance to carbapenems. In strain S04 90, but not in Carb01 63, the *mexZ* gene (locus tag YH69_17355) is disrupted by a frameshift mutation. Inactivation of MexZ leads to overproduction of the MexXY components of the MexXY-OprM efflux pump and is associated with increased resistance to aminoglycosides, fluoroquinolones, and zwitterionic cephalosporins, such as cefepime, amongst others (Guénard et al., 2014). Both strains carry missense mutations leading to a T83I amino-acid substitution in the GyrA protein and an S87L substitution in ParC, which are associated with resistance to fluoroquinolones (Kos et al., 2015). These mutations explain the observed resistance of the strains to ciprofloxacin (**Table 1**).

## Discussion

The two *P. aeruginosa* strains analyzed here belong to two MBL-producing clones that are endemic in Dutch hospitals (Van der Bij et al., 2011). Carb01 63 is a representative of the most prevalent clone. The spread of this ST111 clone among various hospitals in The Netherlands has been demonstrated (Van der Bij et al., 2012), and this type caused the first outbreak (Van der Bij et al., 2011). Carb01 63, which was isolated from drains and sinks in the Maasstad hospital, is closely related to the recently sequenced strain RIVM-EMC2982 (accession number CP016955.1), a patient isolate from the Erasmus University Medical Center in Rotterdam. Carb01 63 is also closely related to previously described outbreak strains of the same ST111 and O12 antigen, reported in hospitals in the United Kingdom (Breathnach et al., 2012; Witney et al., 2014; Turton et al., 2015). The most closely related neighbors of strain S04 90 are two clinical ST446 isolates from France, strains AZPAE15043 (Kos et al., 2015) and WH-SGI-V-07172 (van Belkum et al., 2015), which are susceptible to carbapenems.

Both strains are resistant to many antibiotics, and the presence of *bla*_V IM-2_ and other resistance genes was demonstrated. The presence of *bla*_V IM-2_ genes in *P. aeruginosa* isolates has been repeatedly described, but how these genes spread among strains is usually not clear because, apart from the cassette composition of the class 1 integrons in which they are located, their genetic context is often not described. In *P. aeruginosa, bla*_V IM-2_-containing integrons are usually associated with the chromosome as was found in strain Carb01 63, but it is present on a plasmid in strain S04 90. Although plasmid-associated *bla*_V IM-2_ genes have occasionally been reported before (Poirel et al., 2000; Edelstein et al., 2013; Wright et al., 2015), only in a single case the gene was shown to be present on a conjugative plasmid (Botelho et al., 2017). We have experimentally demonstrated that also pS04 90 is a conjugative plasmid. The plasmid shows high similarity to pND6-2, a previously described plasmid from *P. putida* strain ND6 that was suggested to belong to a new plasmid incompatibility (Inc) group (Li et al., 2013). Nucleotide BLAST searches showed that pS04 90 has high sequence similarity to pND6-2 also in the DNA fragment containing the *oriV* and the *repB* gene, suggesting that pS04 90 belongs to this same new Inc group. DNA segments with high sequence similarity to the plasmid backbone were also found on contigs of several other incomplete *P. aeruginosa* genome sequences (see **Supplementary Figure S4** for an example), indicating that the plasmid is more commonly found in this species. As compared with pND6-2, pS04 90 has acquired a large DNA fragment of ∼30 kb that contains the integron with the *bla*_V IM-2_ gene. Interestingly, the *bla*_V IM-2_-containing integron is located on a very similar DNA fragment on the chromosome of strain Carb01 63 that, apart from the absence of the IS*222* element and the incomplete IRR of the severely disrupted Tn*4661*, deviates from the DNA fragment of pS04 90 in the presence of only 42 SNPs over the entire 29,963 nt sequence. Also the closely related strain RIVM-EMC2982 contains this DNA fragment on the chromosome in the same position as in Carb01 63, and it is even more closely related to that on pS04 90 with only 5 SNPs besides the absence of the IS*222* element and the incomplete IRR. Thus, it appears that the entire ∼30-kb DNA fragment can move position between plasmid and chromosome as a novel, composite transposon, and, in view of the very high sequence identity of these transposons, this could have occurred only very recently. Since pS04 90 is a conjugative plasmid, this illustrates how MBL-encoding integrons can be mobilized and transferred between strains. Interestingly, the entire composite transposon also appears to be present in a *P. aeruginosa* isolate of Czech origin, i.e., strain Pae-31448cz (Papagiannitsis et al., 2017). However, the severely disrupted Tn*4661*, although present in the available nucleotide sequence (Genbank accession number KY860571.1), was not noticed and, therefore, the possibility that entire fragment could function as a composite mobile element was not considered.

Besides the *bla*_V IM-2_-containing integron, strain Carb01 63 contains an additional class 1 integron containing *aacA4, bla*_CARB-2_, and *aadA2* gene cassettes. The integron is located on a composite transposon that is integrated into an ICE. Apart from the cassette composition of the integron, this ICE is very similar to PAGI-16 in the Korean isolate KMU11 (Hong et al., 2016), probably reflecting a common origin of these genomic islands. The presence of the island in strains of different sequence types, i.e., ST111 and ST235 for Carb01 63 and KMU11, respectively, suggests that it may be transferred between strains. Interestingly, in strain RIVM-EMC2982, which is highly related to Carb01 63, the ICE is split into two parts located at different chromosomal positions and each associated with an IS*6100* element. Similarly, PAGI-16 has been reported to be split into two parts in Korean strain BP14 by a large chromosomal inversion resulting from duplication and insertion of the IS*6100* element (Hong et al., 2016). A similar recombination event apparently occurred in strain RIVM-EMC2982, where it disrupted the *oprD* gene, which encodes a porin mediating transport of carbapenems across the outer membrane.

In our hospitals, MBL-producing clones of ST111 are persistent in sinks and drains despite treatment with 10% hypochlorite, and they are spreading to different departments. These unpublished findings oppose previous results that showed successful reduction of extensively drug-resistant strains from these systems (Witney et al., 2014). Further studies into the resistance of the strains to disinfectants will be facilitated by the available genome sequences. The *P. aeruginosa* strains studied are susceptible only to colistin treatment. Recent discovery of widespread colistin resistance in bacteria urges to keep alert of possible colistin resistance of already extensively drug-resistant *Pseudomonas*, which is a major concern for hospitals, since no option is available for adequate disinfection of drains and sinks, while colonization, infection and outbreaks via these routes are difficult to control.

## Conclusion

The genome sequences of two multidrug-resistant *P. aeruginosa* strains endemic in Dutch hospitals revealed the presence of a novel, large, composite transposon that carries a class 1 integron with a *bla*_V IM-2_ gene and aminoglycoside-resistance genes. This transposon can apparently move position between chromosome and a conjugative plasmid, with which it can be transferred to other *P. aeruginosa* strains. Besides, the genome sequences revealed other mobile resistance genes and mutations in the core genome that contribute to the multidrug-resistance phenotype.

## Author Contributions

AZ, JO, and WG initiated the study. WK and AB performed the experimental work. WP performed the whole genome sequencing and assembly. WK, AZ, and JT performed the bioinformatic analysis and drafted the manuscript. All authors read and approved the final manuscript.

## Conflict of Interest Statement

The authors declare that the research was conducted in the absence of any commercial or financial relationships that could be construed as a potential conflict of interest.

## Footnotes

1 http://www.federa.org

2 http://www.ncbi.nlm.nih.gov/

3 http://pubmlst.org/paeruginosa/

4 http://www-is.biotoul.fr

5 http://www.ncbi.nlm.nih.gov/genome/187
