# Supplementary material for: Spread of Carbapenem Resistance by Transposition and Conjugation Among Pseudomonas aeruginosa
Source: Front Microbiol. 2018 Sep 5;9:2057. doi: 10.3389/fmicb.2018.02057 (PMC6133989; doi:10.3389/fmicb.2018.02057)
Supplement: Supplementary file 1 [file Data_Sheet_1.PDF]

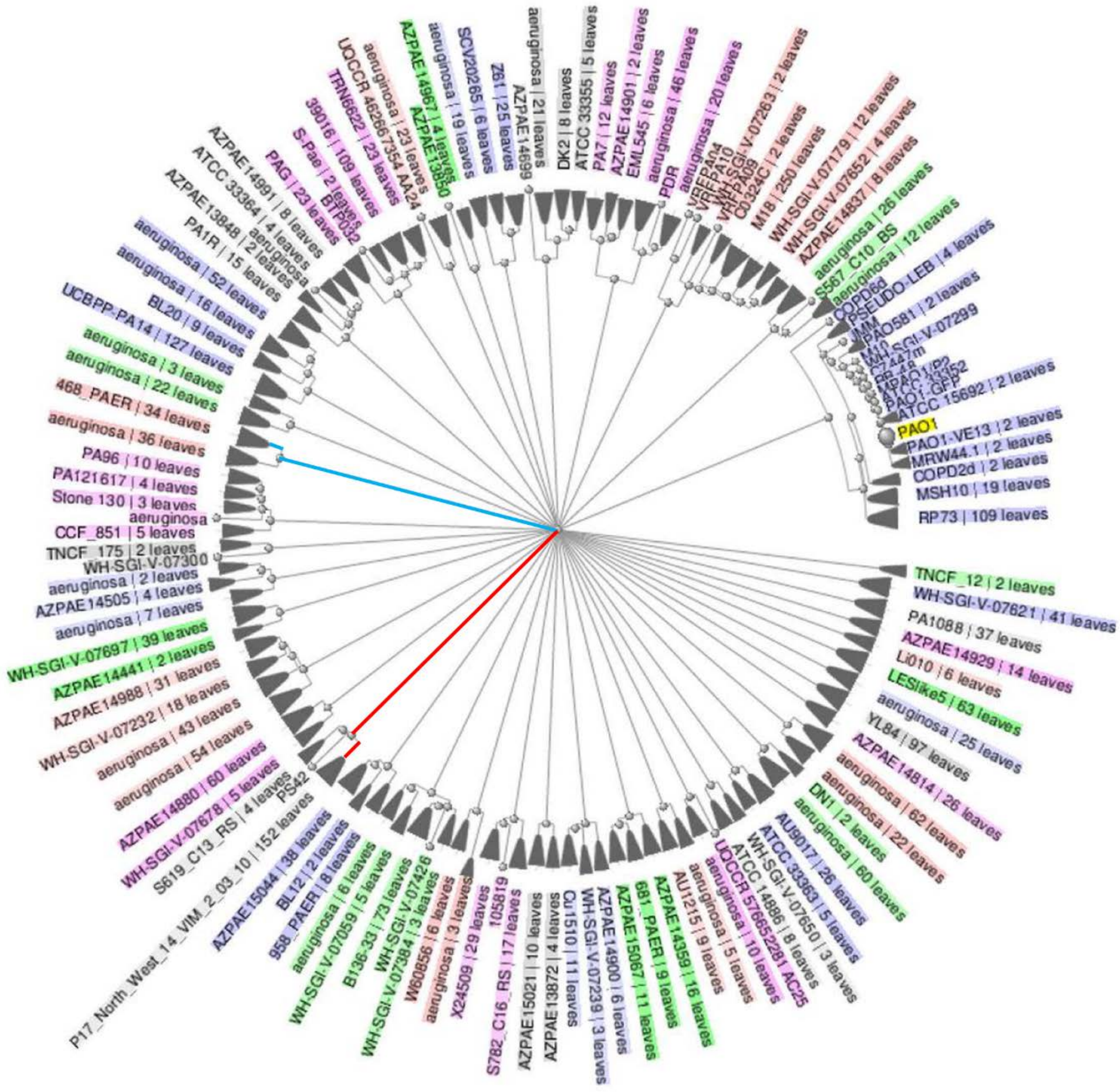

Figure S1 | Dendrogram of genomic BLAST. Carb01 63 belongs to a large clade of 152 leaves (red line), which is represented by P17_North_West_14_VIM_2_03_10. Within this clade, its genome sequence showed 99% identity with query coverage of 99% with that of strain RIVM-EMC2982, isolated in the Erasmus University Medical Center in Rotterdam. S04 90 belongs to a different clade of 34 leaves (blue line), which is represented by 468_PAER.
