# Supplementary material for: Spread of Carbapenem Resistance by Transposition and Conjugation Among Pseudomonas aeruginosa
Source: Front Microbiol. 2018 Sep 5;9:2057. doi: 10.3389/fmicb.2018.02057 (PMC6133989; doi:10.3389/fmicb.2018.02057)
Supplement: Supplementary file 2 [file Data_Sheet_2.PDF]

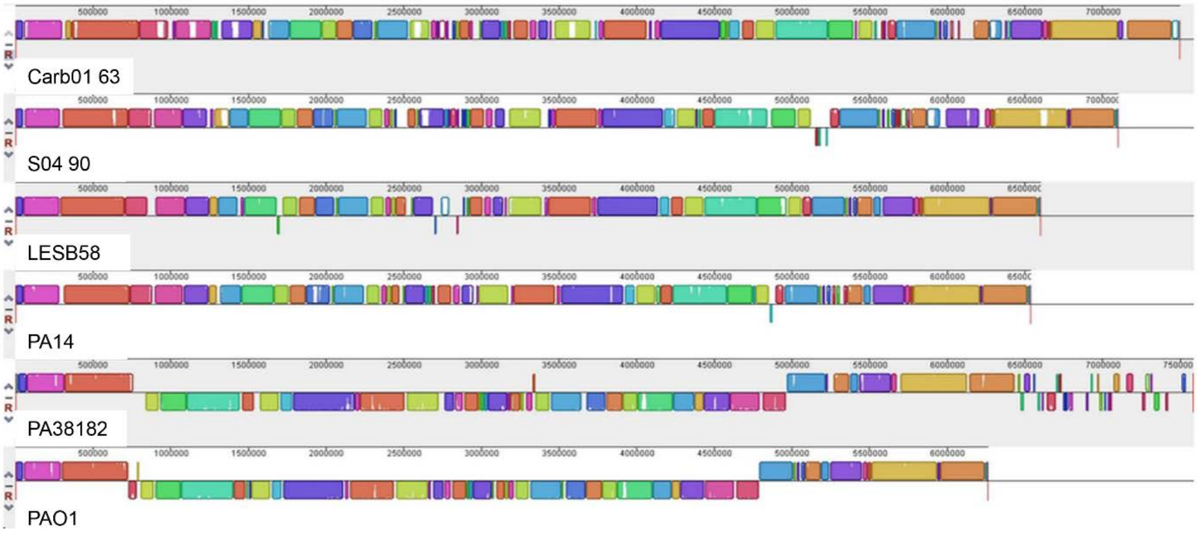

Figure S2 | Alignment of whole genome sequences of strains Carb01 63, S04 90, and some well characterized *P. aeruginosa* genome sequences. Schematic representations of the genome sequences of Carb01 63, S04 90, LESB58 (Winstanley et al., 2009), PA14 (Lee et al., 2006), PA38182 (Witney et al., 2014), and PAO1 (Stover et al., 2000) were made by progressive Mauve. Identical and full colored bars represent homologous regions. White spaces represent the regions with no homology between the sequences used in this comparison. The identities of the genome sequences and the scale of the base pairs are shown below and above the schematics, respectively. Sequence identities of Carb01 63 and S04 90 to PAO1, LESB58, PA38182 and UCBB-PA14 were all 99% with query coverage of 81 and 85%, 85 and 88%, 95 and 88%, and 84 and 87%, respectively. Note that the Carb01 63 genome sequence shows in this comparison the highest similarity (99% identity with 95% coverage) with that of strain PA38182, which is also a multidrug-resistant ST111 serotype O12 strain.

References

Lee, D. G., Urbach, J. M., Wu, G., Liberati, N. T., Feinbaum, R. L., Miyata, S., et al. (2006). Genomic analysis reveals that *Pseudomonas aeruginosa* virulence is combinatorial. *Genome Biol.* 7:R90. doi: 10.1186/gb-2006-7-10-r90

Stover, C. K., Pham, X. Q., Erwin, A. L., Mizoguchi, S. D., Warrener, P., Hickey M. J., et al. (2000). Complete genome sequence of *Pseudomonas aeruginosa* PAO1, an opportunistic pathogen. *Nature* 406, 959-964. doi: 10.1038/35023079

Winstanley, C., Langille, M. G. I., Fothergill, J. L., Kukavica-Ibrulj, I., Paradis-Bleau, C., Sanschagrin, F., et al. (2009). Newly introduced genomic prophage islands are critical determinants of in vivo competitiveness in the Liverpool Epidemic Strain of *Pseudomonas aeruginosa*. *Genome Res* 19, 12-23. doi: 10.1101/gr.086082.108

Witney, A. A., Gould, K. A., Pope, C. F., Bolt, F., Stoker, N. G., Cubbon, M. D., et al. (2014). Genome sequencing and characterization of an extensively drug-resistant sequence type 111 serotype O12 hospital outbreak strain of *Pseudomonas aeruginosa*. *Clin. Microbiol. Infect.* 20, O609-O618. doi: 10.1111/1469-0691.12528
