# Supplementary material for: Spread of Carbapenem Resistance by Transposition and Conjugation Among Pseudomonas aeruginosa
Source: Front Microbiol. 2018 Sep 5;9:2057. doi: 10.3389/fmicb.2018.02057 (PMC6133989; doi:10.3389/fmicb.2018.02057)
Supplement: Supplementary file 3 [file Data_Sheet_3.PDF]

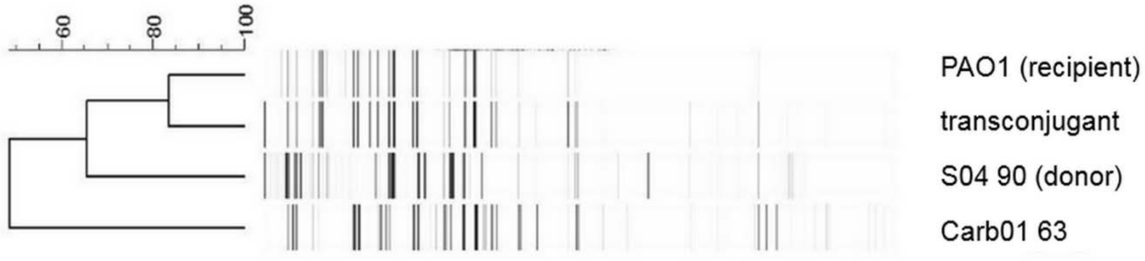


**Figure S3 | Cluster analysis of AFLP typing patterns of donor strain S04 90, recipient strain PAO1, and one of the transconjugants obtained**. For comparison, also the pattern of Carb01 63 is included.
