# Supplementary material for: Spread of Carbapenem Resistance by Transposition and Conjugation Among Pseudomonas aeruginosa
Source: Front Microbiol. 2018 Sep 5;9:2057. doi: 10.3389/fmicb.2018.02057 (PMC6133989; doi:10.3389/fmicb.2018.02057)
Supplement: Supplementary file 4 [file Data_Sheet_4.PDF]

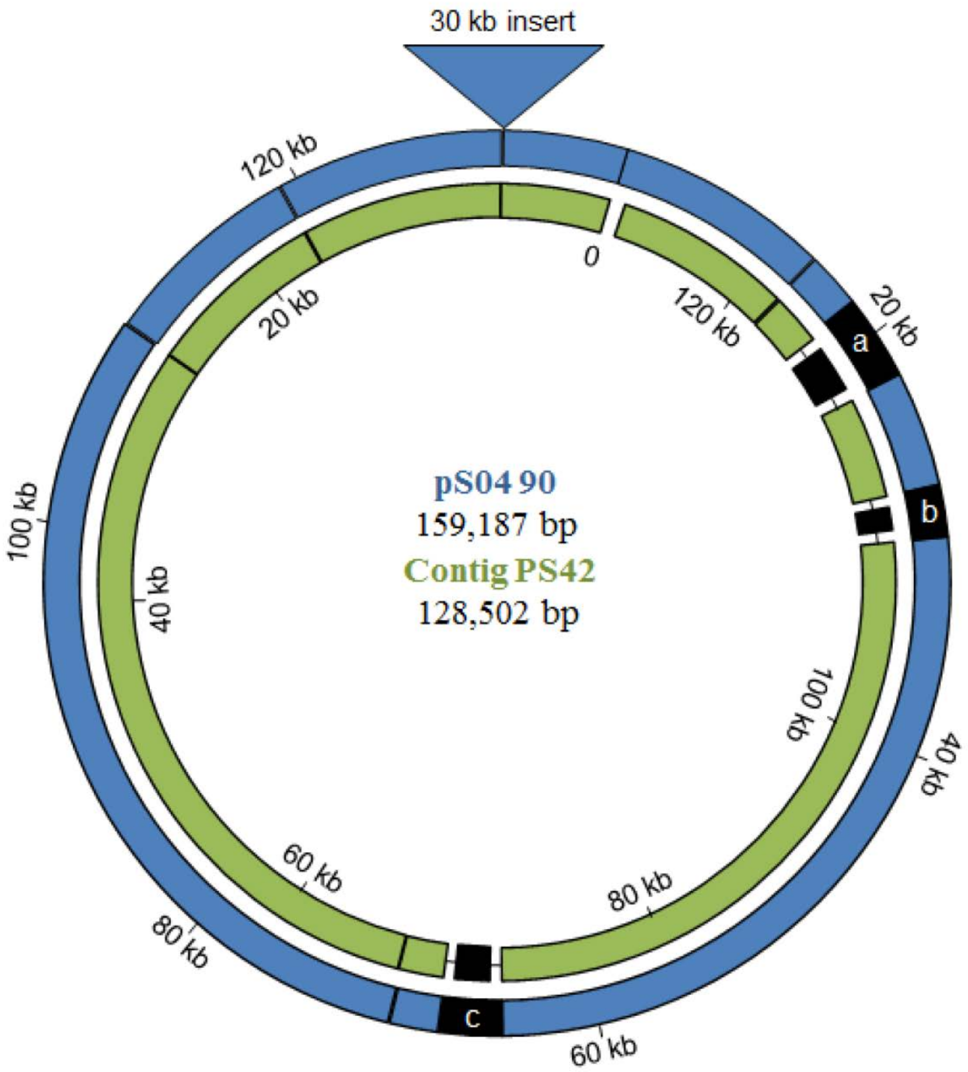

Figure S4 | Presence of the pS04 90 plasmid backbone in other *P. aeruginosa* strains. BLAST searches revealed the presence of pS04 90-releated sequences on contigs of several incompletely finished *P. aeruginosa* genome sequences. As an example, a comparison of pS04 90 (blue) with a 128,502-bp contig (accession number AZZD01000055.1) of *P. aeruginosa* strain PS42 (green) is shown. Regions with >99% sequence identity are indicated by color. Non-homologous regions are black and deletions are indicated by a line. Besides the absence of the 30-kb composite transposon carrying the integron with the $bla_{\mathrm{VIM\text{-}2}}$ gene in the PS42 contig, there are three divergent regions in the aligned sequences marked a, b, and c. Segment a of pS04 90 contains a 2606-bp IS element of the IS*21* family with 99% similarity to IS*Pst3* originally found in *Pseudomonas stutzeri*, an XRE family transcription regulator, and a toxin-antitoxin addiction module designated DinJ-YafQ. Segment b contains only small putative genes encoding hypothetical proteins, and segment c contains the *oriV* and the *repB* gene that are replaced by a different replication module in PS42.
